# Construction and Comprehensive Analysis of miRNAs and Target mRNAs in *Longissimus dorsi* Muscle of Queshan Black and Large White Pigs

**DOI:** 10.3390/life12111814

**Published:** 2022-11-07

**Authors:** Xuelei Han, Kunlong Qi, Chenglei Song, Yaqing Dou, Yingke Liu, Chenlei Li, Yilin Wei, Ruimin Qiao, Xiuling Li, Feng Yang, Kejun Wang, Xinjian Li

**Affiliations:** College of Animal Science and Technology, Henan Agricultural University, Zhengzhou 450046, China

**Keywords:** Queshan Black pigs, Large White pigs, pork quality, RNA-seq, differentially expressed, miRNA

## Abstract

**Simple Summary:**

In this study, the miRNA-mRNA combination analysis of the longissimus dorsi of adult Queshan Black and Large White pigs was carried out by RNA-seq technology, and the molecular mechanism affecting pork quality traits was revealed. The results showed that 39 miRNAs were differentially expressed between Queshan Black and Large White pigs. A functional enrichment analysis of their target genes showed that they were mainly enriched in pathways related to adipogenesis and cell proliferation and differentiation. In addition, miR-328, as a key miRNA, can affect the proliferation of 3T3-L1 cells. This study revealed the miRNA-mRNA regulatory axis affecting fat deposition and skeletal muscle mass and provided a theoretical basis for further study of the molecular regulation mechanism of meat quality traits.

**Abstract:**

A miRNA-mRNA combination analysis was performed on the longissimus dorsi muscle of adult Queshan Black and Large White pigs by RNA-seq technology to reveal the molecular mechanism affecting pork quality traits. The sequencing results showed that 39 miRNAs were differentially expressed between Queshan Black and Large White pigs, which targeted 5234 mRNAs, and 15 differentially expressed miRNAs targeted 86 differentially expressed mRNAs. The qRT-PCR results showed that miRNAs showed similar expression patterns to RNA-seq. The GO analysis indicated that differentially expressed miRNAs with differential target mRNAs were primarily involved in biological processes such as phospholipase activity, MAP-kinase scaffold activity, lipase activity, and regulation of the extent of cell growth. The KEGG analysis also revealed that such mRNAs were significantly enriched in the ECM-receptor interaction, sphingolipid metabolism, apoptosis, PI3K-Akt signaling pathway, and AMPK signaling pathway. In addition, software predictions showed that 17 (13 of which were upregulated and four were downregulated) of 39 differentially expressed miRNAs targeted 118 negatively correlated expression mRNAs. The upregulated miRNAs contained 103 negatively correlated target mRNAs, whereas the downregulated miRNAs contained 15 negatively correlated target mRNAs. The GO analysis showed that such mRNAs were primarily involved in MAP-kinase scaffold activity, myoblast development, and peptidyl-lysine methylation, and the KEGG analysis showed significant enrichment in ECM-receptor interaction and focal adhesion. The functional enrichment analysis of miRNA target genes revealed that miR-328 was screened out as a key miRNA, and preliminary functional validation was performed. Moreover, the overexpressed miR-328 could affect the expression of proliferation-related genes, such as CDK2, CDK4, CCNB1, CCND1, CCNE1, and PCNA. These results indicated that miR-328 may regulate fat deposition and affect meat quality by influencing related pathways. This study revealed that the miRNA−mRNA regulatory axis affects fat deposition and skeletal muscle development, which provides a theoretical basis for further study on the molecular mechanism of meat quality.

## 1. Introduction

Pork is an essential and major source of animal protein for humans. In recent years, the quality of pork has become more important than the quantity because of the improvement in people’s living standards. In addition, pigs are often used as a model to understand human biology because of their physiology and anatomical similarities to humans [1,2]. Pork quality is closely related to intramuscular fat content, drip loss, tenderness, shear force, and meat color, which is an important economic trait in pig husbandry, with high-quality meat products being an important goal in livestock production. Significant differences in growth rate, muscle mass, and lipid deposition capacity are observed among different breeds [3]. Elucidating the molecular basis of fat deposition in pigs is important to provide a comprehensive understanding of diseases associated with fat metabolism. However, the mechanisms regulating pork quality remain unclear.

MicroRNAs (miRNAs) are small, and siRNA-like molecules are important in the epigenetic regulation of gene expression. Mature miRNAs are approximately 21−24 nucleotides in length, and they can bind to argonaute proteins to form miRNA-induced silencing complexes [4]. The seed sequence of miRNA is a segment of seven nucleotides spanning 2−8 nucleotides at the 5′ end of the miRNA, which usually regulates the expression of the target gene by binding to its 3′-untranslated regions [5]. However, miR-10a also interacts with the 5′ untranslated region of mRNAs encoding ribosomal proteins to enhance their translation [6]. miRNAs are quite conserved in species evolution, and those found in plants, animals, and fungi are only expressed in specific tissues and developmental stages. In addition, their tissue specificity and temporal sequence determine tissue and cellular functional specificity, indicating that miRNAs play different roles in the regulation of cell growth, development processes, and meat quality. Muscle-specific miR-1 and miR-133, which play important roles in muscle growth and differentiation, manipulate the signaling pathways that control MEF2 activity to regulate the expression of miR-1 and miR-133 [7]. The ectopic expression of miR-103 or miR-143 in preadipocytes accelerates adipogenesis [8]. Based on previous studies, the knockdown of miRNA378 and/or miRNA378* can reduce the accumulation of triacylglycerols, and miRNA378/378* specifically increases the transcriptional activity of C/EBPα and C/EBPβ on adipocyte gene promoters [9]. miR-17-92 can promote adipocyte differentiation by targeting and negatively regulating Rb2/p130 [10]. let-7 plays an important role in adipocyte differentiation by targeting HMGA2. Therefore, let-7 may be important in obesity and other forms of metabolic disease, in which the amount and/or function of adipose tissue changes [11]. miR-181 can target Hox-A11 to regulate mammalian myoblast differentiation [12].

As a domestic breed in China, the Queshan Black pig has excellent meat quality and strong stress resistance, which would be an excellent model to investigate the differences in meat quality between native and exotic breeds. In a previous study, we analyzed and compared the differences in immune traits between Queshan Black and Large White pigs using RNA-seq [13]. In this study, we obtained miRNA expression changes between the longissimus dorsi of Queshan Black and Large White pigs by transcriptome sequencing to explore the miRNAs influencing meat quality. The results of this study identified potential miRNA-mRNA associated with pork fat deposition and skeletal muscle development and indicated their potential roles in muscle phenotypic differences among different pig breeds. These results lay the foundation for further studies on the molecular mechanism of meat quality.

## 2. Materials and Methods

### 2.1. Tissue Collection and RNA Extraction

Sample collection has been described previously; the animal samples in this study were derived from three Queshan Black pigs and three Large White pigs of the same nutritional level and weight. The experimental animals were free to feed and drink water, and lived under the same normal conditions, fasting the night before slaughter [14]. In brief, Queshan Black and Large White pigs were fasted and humanely slaughtered the next day, and the longissimus dorsi at the 6–7th rib of the right side of the carcass was collected, frozen in liquid nitrogen, and stored at −80 °C until RNA extraction. Total RNA was extracted from the samples using a TRIzol reagent (15596026, Thermo Fisher Scientific, Waltham, MA, USA). Agarose gel (1%); a NanoPhotometer^®^ Spectrophotometer (IMPLEN, Westlake Village, CA, USA); Qubit^®^ RNA Assay Kit in Qubit^®^ 2.0 Fluorometer (Life Technologies, Carlsbad, CA, USA); Agilent Bioanalyzer 2100 System (Agilent Technologies, Santa Clara, CA, USA); and RNA Nano 6000 Assay Kit were used to verify RNA concentration and quality.

### 2.2. RNA Sequencing and Data Analysis

An RNA library was constructed for each breed using the total RNA of three individuals from Queshan Black and Large White pig, respectively. The library construction and sequencing procedures were performed as previously described. In brief, a sequencing library was generated using the NEBNext^®^ Multiplex Small RNA Library Prep Set for Illumina^®^ (NEB, Ipswich, MA, USA) according to the manufacturer’s recommendation, and index codes were added to each attribute sequence of the sample. First-strand cDNA was synthesized using M-MuLV reverse transcriptase (RNase H-). PCR amplification was performed using LongAmp Taq 2× Master Mix, SR Primer for Illumina, and index (X) primers. PCR products corresponding to 140–160 bp were recovered and dissolved in elution buffer. Finally, library quality was assessed on an Agilent Bioanalyzer 2100 system using high-sensitivity DNA chips. Index-encoded samples were clustered on the cBot Cluster Generation System using the TruSeq SR Cluster Kit v3-cBot-HS (Illumina) according to the manufacturer’s instructions. After cluster generation, library preparations were sequenced on the Illumina Hiseq 2500 platform, and 50 bp single-end reads were generated. The raw data were evaluated, and low-quality reads were removed (reads containing ploy-N, with 5′ adapter contaminants, without 3′ adapter or the insert tag, containing ploy A or T or G or C and low-quality reads). After the quality control of raw reads, the quality of information analysis was ensured; clean reads were obtained, and only these were used for subsequent analysis.

### 2.3. Enrichment Analysis of Differential miRNA Target Genes

The intersection of miRanda, PITA, and RNAhybrid is the final target gene prediction result. After obtaining differentially expressed miRNAs, Gene Ontology (GO) [15] and Kyoto Encyclopedia of Genes and Genomes (KEGG) [16] enrichment analyses were performed on the basis of the corresponding relationship between miRNAs and target genes. KEGG is the main public database on the pathway. The most important biochemical metabolic and signal transduction pathways involved in candidate target genes can be determined by pathway significant enrichment analysis. GO and KEGG analyses were performed using OmicShare tools (https://www.omicshare.com/tools, accessed on 11 June 2022).

### 2.4. Quantitative Real-Time PCR (qRT-PCR)

Approximately 1 μg of each RNA sample was used in the PrimeScript™ RT reagent Kit with gDNA Eraser (Perfect Real Time; Code No. RR047A, Takara, Beijing, China) to convert total RNA to cDNA, with random hexamers (for mRNA) and Bulge-Loop U6 qRT-PCR Primer Set for miRNAs (RN: R10031.8, RiboBio, Guangzhou, China) according to the manufacturer’s instructions. Next, a qRT-PCR was performed using TB Green^®^ Premix Ex Taq™ II (Code No. RR820A, Takara, Beijing, China) on the CFX96 Real Time PCR Detection System (Thermo Fisher Scientific, USA). The reaction program was as follows: denaturation at 95 °C for 3 min, followed by 45 cycles at 95 °C for 10 s and 60 °C for 30 s. Meanwhile, GAPDH (glyceraldehyde-3-phosphate dehydrogenase, for mRNA) and U6 (for miRNA) were used as normalization controls, and all reactions were carried out in triplicate. The 2^−ΔΔCT^ method was used to calculate the relative gene expression level.

### 2.5. 3T3-L1 Cell Culture and Treatment

3T3-L1 murine preadipocytes were cultured in Dulbecco’s Modified Eagle Medium supplemented with 10% (*v*/*v*) FBS until confluence. Gema Pharmaceutical Technology (Shanghai, China) synthesized the miRNA mimics and inhibitor. The cells were transfected by Lipofectamine 3000 Reagent (Invitrogen, Carlsbad, CA, USA) according to the manufacturer’s instructions. RNA was harvested 48 h after transfection.

### 2.6. Data Accessibility

All raw data of high-throughput sequencing were deposited to the National Genomics Data Center (NGDC, https://bigd.big.ac.cn, accessed on 19 September 2021) with the dataset accession number CRA005025.

### 2.7. Statistical Analysis

R (version 4.2.0) was used for the statistical analysis. The data obtained from this study were presented as mean ± SEM. Differences among groups were analyzed using a Student’s *t*-test. *p* < 0.05 was regarded as statistically significant.

## 3. Results

### 3.1. Overview of Small-RNA Sequencing Data

**Figure 1:**
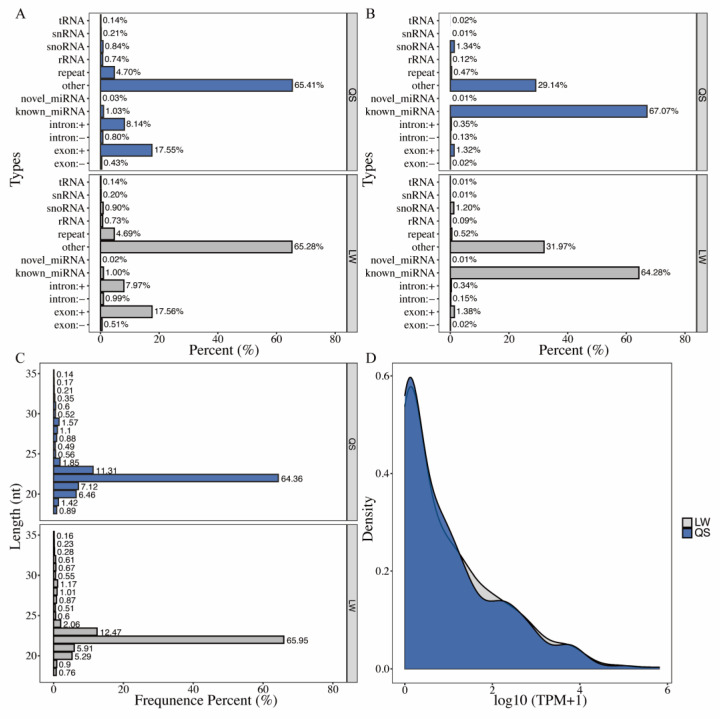
Overview of small-RNA sequencing data. (**A**) Statistics of various types of sRNAs on comparison; (**B**) statistics of the number of various types of sRNAs on comparison; (**C**) statistics of the length distribution of the obtained total sRNA fragments; (**D**) TPM density distribution of miRNA expression. Note: QS represents Queshan Black pig, LW represents Large White pig.

By Illumina HiSeq 2500 sequencing, an average of 30,495,011 and 25,844,213 raw reads were generated from the longissimus dorsi muscle of adult Queshan Black and Large White pigs, respectively. After removing adapters, contamination, and low-quality reads, 29,825,036 and 25,453,427 clean reads were obtained (Table 1). Approximately 97.81% and 98.48% of the clean reads were mapped to the pig reference genome (version Sscrofa11.1/susScr11, Table 2). A small RNA library was constructed using the total RNA of the longissimus dorsi of Queshan Black pigs and Large White pigs to decipher the characteristics of miRNAs in the longissimus dorsi of different pig species. After removing small RNAs matching rRNA, tRNA, snRNA, snoRNA, or scRNA, a total of 102,179,331 (65.79%) known miRNAs and 11,214 (0.01%) novel miRNAs were obtained in Queshan Black and Large White pigs, respectively (Figure 1).

In the present study, we categorized and annotated small RNAs and summarized all small RNAs with various types of RNAs for comparison and annotation. One sRNA was simultaneously compared with several pieces of annotation information to obtain a unique annotation for each unique sRNA; hence, small RNAs were annotated on the basis of the known miRNA > rRNA > tRNA > snRNA > snoRNA > repeat > NAT-siRNA > gene > novel miRNA > ta-siRNA. The small RNAs were traversed in priority order. The total number of rRNAs in the classification annotation results can be used as a quality control standard for a sample. The results showed that the number of rRNAs in the samples of Queshan Black and Large White pigs were 0.12% and 0.09%, respectively, whereas their rRNA species were 0.74% and 0.73%, indicating that the sequencing samples were of high quality (Figure 1A,B). The clean reads of each sample of Queshan Black and Large White pigs were screened for sRNAs in a certain length range for subsequent analysis, and the length distribution statistics of sRNAs was obtained (Figure 1C). In general, the length range of animal sRNAs was 18–35 nt, and the length of the sRNAs sequenced in this study was concentrated at 22 nt. The TPM density distribution was also counted in this study (Figure 1D).

### 3.2. Target mRNAs Prediction of miRNAs

The intersection of miRanda, PITA, and RNAhybrid was used to perform target gene prediction for the known and novel miRNAs obtained from the analysis and to obtain reliable target gene prediction results, and the correspondence between miRNAs and target genes was obtained (Table 3). The results revealed that 418 miRNAs were predicted to yield 10,428 target genes corresponding to 20,375 transcripts, whereas 39 differentially expressed miRNAs with 3064 target genes correspond to 5234 transcripts.

### 3.3. GO and KEGG Annotation of Differentially Expressed miRNAs Targeting mRNA

**Figure 2:**
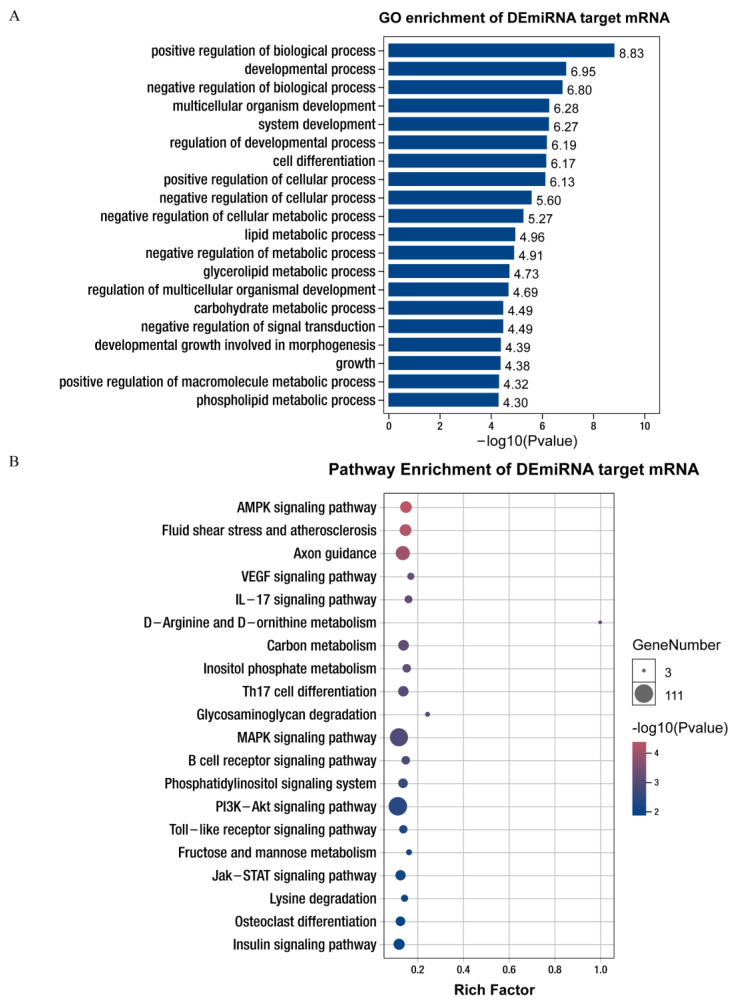
GO and KEGG annotation of differentially expressed miRNA targeting mRNA. (**A**) GO enrichment analysis of potential miRNAs targeting mRNAs of Queshan Black versus Large White pigs. BP: biological process; CC: cell components; MF: molecular function. (**B**) The potential miRNA targeting mRNAs of Queshan Black versus Large White pigs was subjected to pathway enrichment analysis.

The GO enrichment analysis showed that most of the genes in the biological process category (>1000) were significantly enriched in the metabolic process, organic substance metabolic process, primary metabolic process, biological regulation, regulation of biological process, and regulation of cellular processes. The majority of mRNAs in the molecular function category (>200) were significantly enriched during semaphorin receptor binding, beta-galactosidase activity, galactosidase activity, sphingomyelin phosphodiesterase D activity, phosphodiesterase I activity, and MAP kinase activity. Based on the cellular component classification, most mRNAs (>1000) were significantly enriched in cell, cell part, intracellular, intracellular part, organelle, and intracellular organelle (Figure S1, Table S1). In addition, many significantly enriched GO terms were closely related to cellular processes and lipid metabolism, such as the regulation of developmental process, cell differentiation, lipid metabolic process, glycerolipid metabolic process, and phospholipid metabolic process (Figure 2A, Table S2).

The KEGG pathway enrichment analysis revealed that these target mRNAs were annotated to cellular processes (lysosome, focal adhesion, and apoptosis); environmental information processing (ECM; AMPK signaling pathway, MAPK signaling pathway, phosphatidylinositol signaling system, PI3K-Akt signaling pathway, Jak-STAT signaling pathway, and sphingolipid signaling pathway); human diseases (pathways in cancer, AGE-RAGE signaling pathway in diabetic complications); metabolism (D-Arginine and D-ornithine metabolism; inositol phosphate metabolism; glycosaminoglycan degradation; fructose and mannose metabolism; lysine degradation; glycine, serine, and threonine metabolism; and sphingolipid metabolism); and organismal systems (osteoclast differentiation, insulin signaling pathway, and GnRH signaling pathway; Figure S2, Table S3). In addition, many significantly enriched KEGG pathways are closely related to cellular processes and lipid metabolism, such as the AMPK signaling pathway, inositol phosphate metabolism, MAPK signaling pathway, phosphatidylinositol signaling system, PI3K-Akt signaling pathway, Jak-STAT signaling pathway, and lysine degradation. Therefore, these genes may play an important role in regulating intramuscular adipogenesis and lipogenesis in Queshan Black and Large White pigs (Figure 2B, Table S4).

### 3.4. GO and KEGG Annotation and Analysis of Differentially Expressed mRNAs Corresponding to Differentially Expressed miRNAs

**Figure 3:**
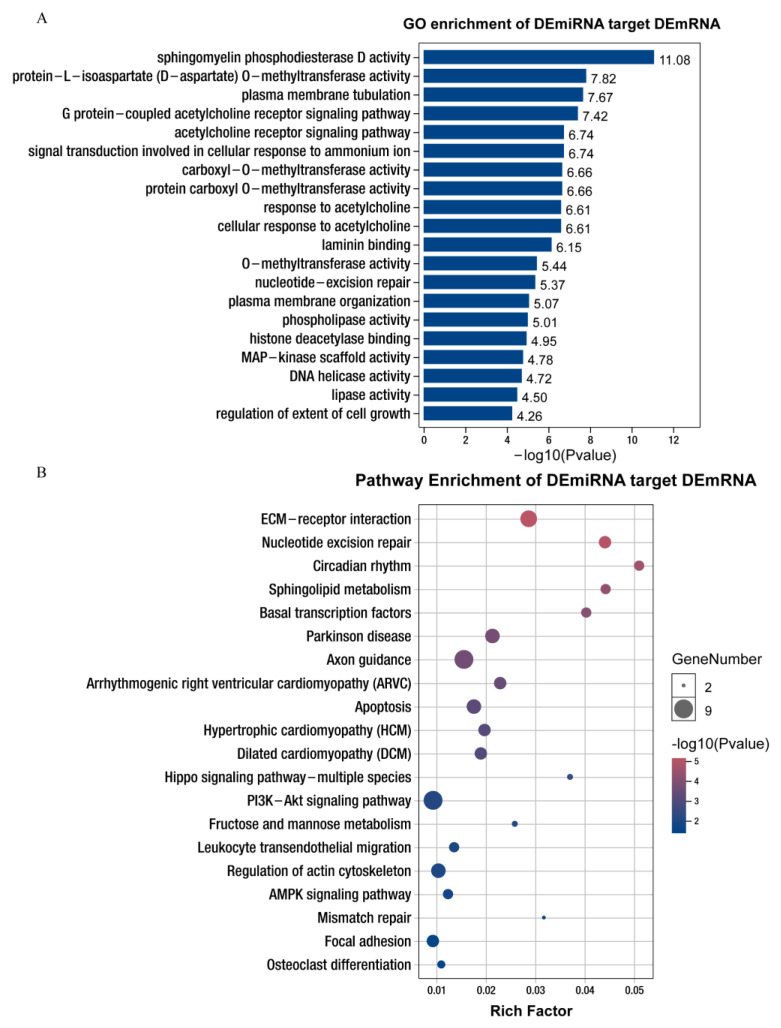
GO and KEGG annotation and analysis of differentially expressed mRNAs corresponding to differentially expressed miRNAs. (**A**) GO enrichment analysis of miRNAs potentially targeting differentially expressed mRNAs of Queshan Black versus Large White pigs. BP: biological process; CC: cell components; MF: molecular function. (**B**) The miRNAs potentially targeting differentially expressed mRNAs of Queshan Black versus Large White pigs were subjected to pathway enrichment analysis.

The GO enrichment analysis showed that most mRNAs (>15) in the biological process class were significantly enriched in cellular process, multicellular organism development, response to chemical, system development, and regulation of multicellular and organismal process. Most of the mRNAs (>25) in the molecular function class were significantly enriched in binding, heterocyclic compound binding, and anion binding. In addition, the majority of mRNAs (>60) were significantly enriched in cell, cell part, and intracellular part (Figure S3, Table S5). Many significantly enriched GO terms are closely related to cellular processes and lipid metabolism, such as sphingomyelin phosphodiesterase D activity, protein-L-isoaspartate (D-aspartate) *O*-methyltransferase activity, phospholipase activity, MAP-kinase scaffold activity, lipase activity, and the regulation of cell growth (Figure 3A, Table S6).

The KEGG pathway enrichment analysis revealed that these target mRNAs were annotated to cellular processes (apoptosis, regulation of actin cytoskeleton, and focal adhesion); environmental information processing (ECM-receptor interaction, Hippo signaling pathway-multiple species, PI3K-Akt signaling pathway, AMPK signaling pathway, HIF-1 signaling pathway, and Ras signaling pathway); genetic information processing (nucleotide excision repair, basal transcription factors, mismatch repair, and DNA replication); human diseases (Parkinson disease, arrhythmogenic right ventricular cardiomyopathy [ARVC], hypertrophic cardiomyopathy, and pathways in cancer); metabolism (sphingolipid metabolism, fructose, and mannose metabolism); and organismal systems (circadian rhythm and osteoclast differentiation; Figure S4, Table S7). In addition, many significantly enriched KEGG pathways are closely associated with cellular processes and lipid metabolism, such as ECM-receptor interaction and sphingolipid metabolism, indicating that these miRNAs may play an important role in the manipulation of adipogenesis through the regulation of genes (Figure 3B, Table S8).

### 3.5. GO and KEGG Annotation and Analysis of Negatively Correlated Target mRNAs of Differentially Expressed miRNAs

miRNAs regulate their function by adsorbing target genes. The results showed that 17 miRNAs, including 13 upregulated and four downregulated, were identified among the 39 differentially expressed miRNAs. In addition, the expression of 118 mRNAs was negatively correlated. Next, we predicted the target mRNAs of these miRNAs, with 103 target mRNAs for 13 upregulated miRNAs and 15 target mRNAs for four downregulated miRNAs in Queshan Black and Large White pigs.

**Figure 4:**
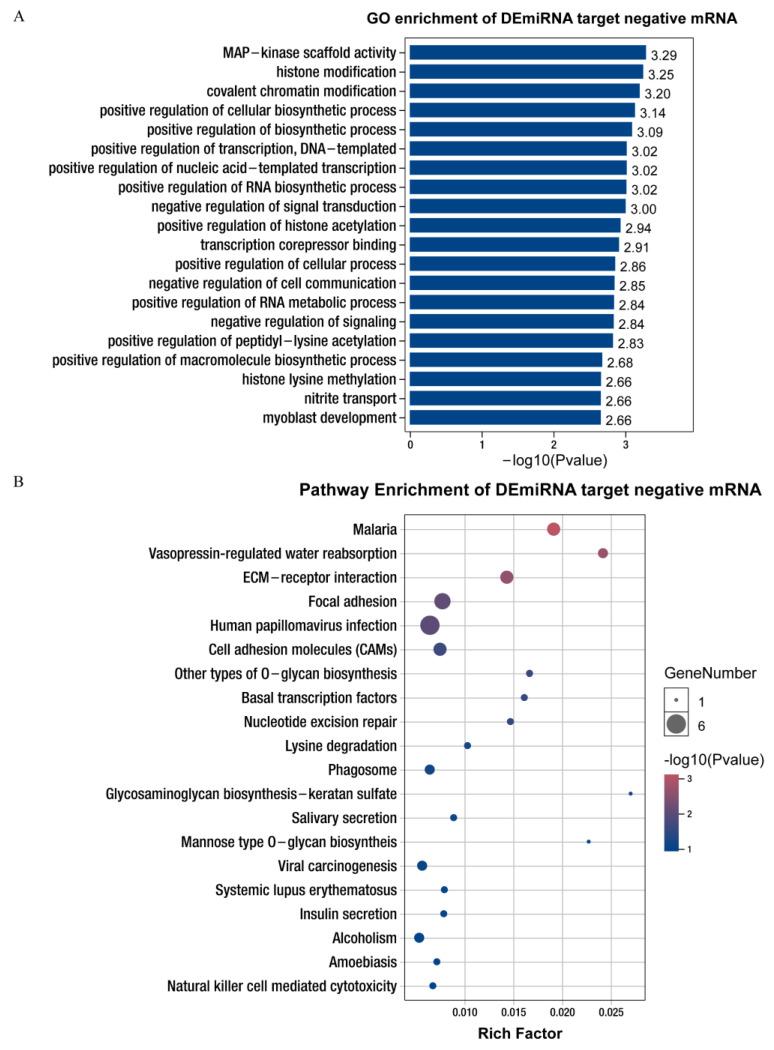
GO and KEGG annotation and analysis of negatively correlated target mRNAs of differentially expressed miRNAs. (**A**) GO enrichment analysis of miRNAs potentially targeting negatively correlated mRNAs of Queshan Black versus Large White pigs. BP: biological process; CC: cell components; MF: molecular function. (**B**) The miRNAs potentially targeting negatively correlated mRNAs of Queshan Black versus Large White pigs were subjected to pathway enrichment analysis.

The GO enrichment analysis showed that most mRNAs (>30) in the biological process class were significantly enriched in biological process, cellular process, metabolic process, organic substance metabolic process, and primary metabolic process. In contrast, most of the mRNAs (>30) in the molecular function class were significantly enriched in molecular function, binding, and protein binding. The majority of mRNAs (>40) were significantly enriched in cellular component, cell, cell part, intracellular, and intracellular part (Figure S5, Table S9). Many significantly enriched GO terms are closely related to cellular and biosynthetic processes, such as the positive regulation of a cellular biosynthetic process, myoblast development, and positive regulation of a biosynthetic process (Figure 4A, Table S10).

The KEGG pathway enrichment analysis revealed that these negative target mRNAs were annotated to cellular processes (focal adhesion); environmental information processing (ECM-receptor interaction, cell adhesion molecules (CAMs)); genetic information processing (basal transcription factors and nucleotide excision repair); human diseases (malaria and human papillomavirus infection); metabolism (other types of *O*-glycan biosynthesis); and organismal systems (vasopressin-regulated water reabsorption; Figure S6, Table S11). In addition, many significantly enriched KEGG pathways are closely associated with cellular processes and lipid metabolism, such as ECM-receptor interaction and focal adhesion, indicating that these miRNAs may play an important role in the manipulation of cell proliferation and differentiation, as well as adipogenesis through gene regulation (Figure 4B, Table S12).

### 3.6. Construction and Analysis of the miRNA−mRNA Network

**Figure 5:**
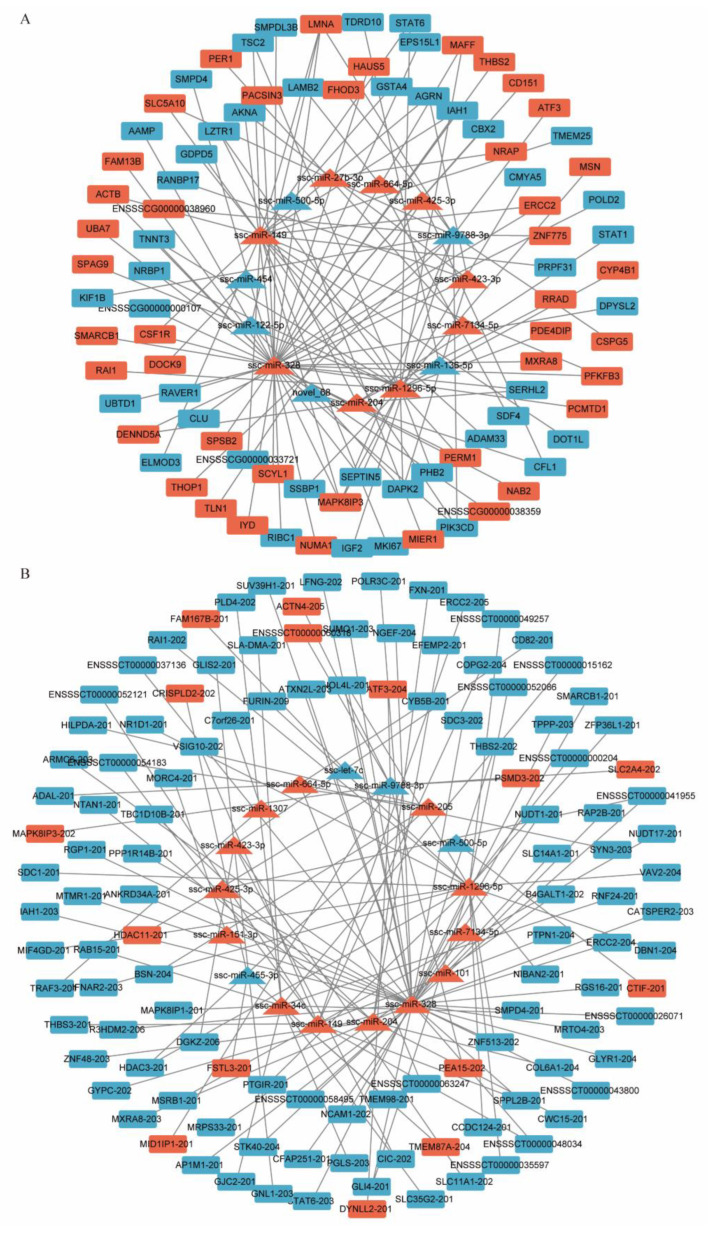
Construction and analysis of the miRNA–mRNA network. (**A**) Regulatory network of differentially expressed miRNAs and differentially expressed mRNAs between Queshan Black and Large White pigs. (**B**) Regulatory network of differentially expressed miRNAs and differentially expressed negatively related mRNAs between Queshan Black and Large White pigs. Red indicates upregulated differentially expressed miRNAs and mRNAs; blue indicates downregulated differentially expressed miRNAs and mRNAs; triangle represents miRNAs, and rounded rectangle represents mRNAs.

This study calculated differentially expressed miRNAs targeting differentially expressed and negatively associated mRNAs in Queshan Black and Large White pigs to construct miRNA−mRNA regulatory networks and identify miRNAs that may play a key role in influencing pork quality. The results showed that 15 miRNAs targeted 86 differentially expressed mRNAs (Figure 5A). Among the differentially expressed miRNAs and targeted negatively associated mRNAs, a total of 17 miRNAs targeting 118 mRNAs were identified (Figure 5B). Combined with the results of the functional enrichment analysis, this study found that miR-149-PIK3CD, miR-204-STAT1, and miR-328-CSF1R/SMPD4 may be the key miRNA−mRNA network affecting skeletal muscle and adipocytes cell proliferation and differentiation, which may affect meat quality.

### 3.7. qRT-PCR Validation of Sequencing Data

**Figure 6:**
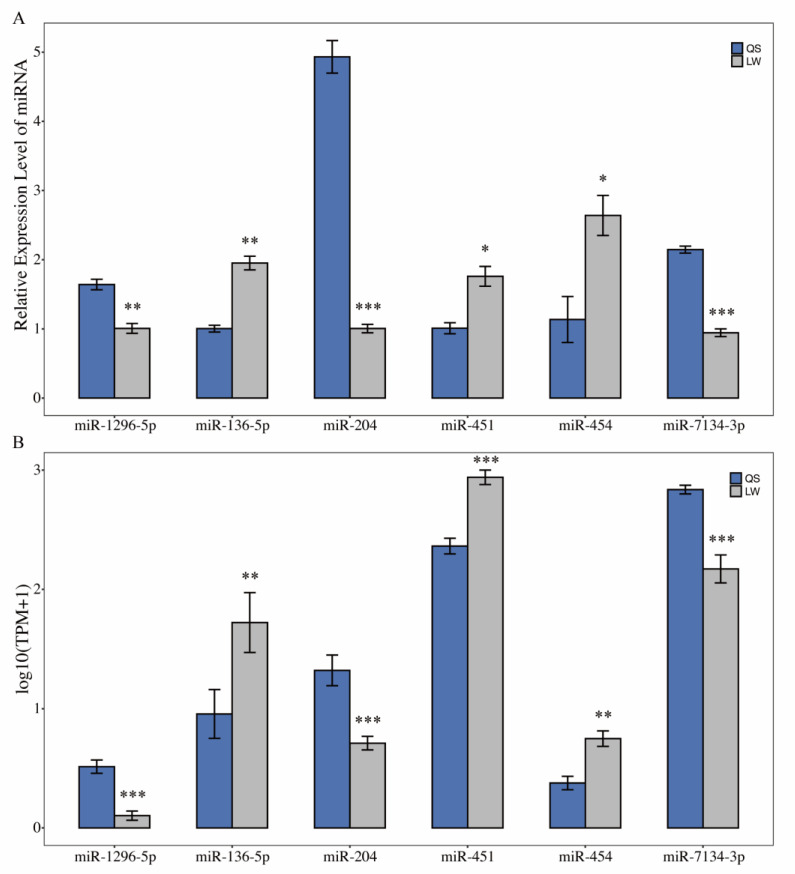
qRT-PCR validation of sequencing data. (**A**) qRT-PCR was performed to detect the expression of six differentially expressed miRNAs (*n* = 3); (**B**) sequencing results of six differentially expressed miRNAs (*n* = 3). Note: QS represents Queshan Black pig; LW represents Large White pig. The U6 (for miRNA) were used as normalization controls; the data represented the Mean ± SEM from 3 biological replicates, and each measurement was repeated 3 times. * indicates *p* < 0.05, ** indicates *p* < 0.01, *** indicates *p* < 0.001.

The expression levels of six differentially expressed miRNAs (miR-1296-5p, miR-136-5p, miR-204, miR-451, miR-454, and miR-7134-3p) were selected and verified by a qRT-PCR to validate the sequencing data. The qRT-PCR and sequencing results indicated that the expression patterns of miRNAs were similar in Queshan Black and Large White pigs (Figure 6). This result further supported the reproducibility and reliability of our sequencing data.

### 3.8. miR-328 Is Negatively Related to the Proliferation of 3T3-L1 Cells

**Figure 7:**
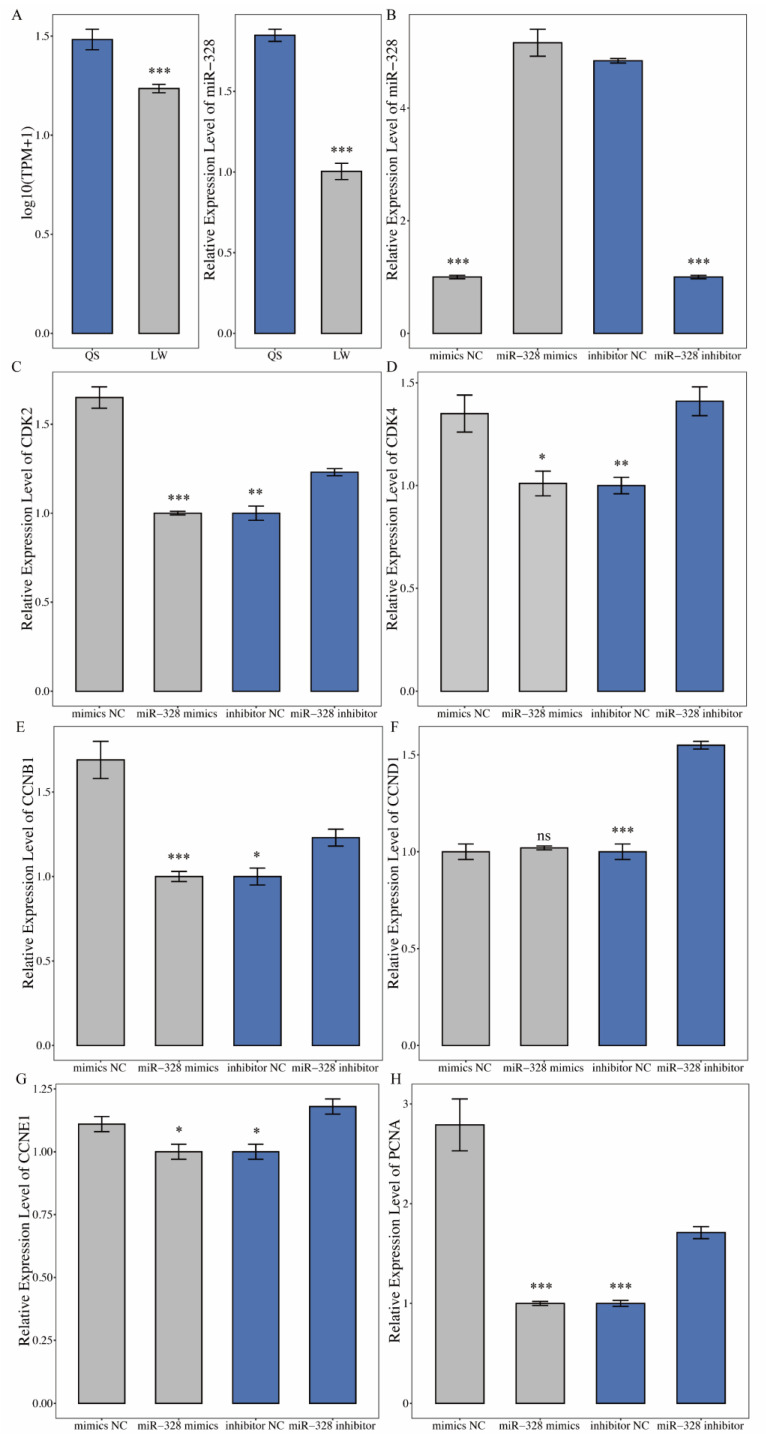
miR-328 is negatively related to the proliferation of 3T3-L1 cells. (**A**) Expression of miR-328 in Queshan Black and Large White pigs, with RNA-seq and qRT-PCR results on the left and right, respectively (*n* = 3). (**B**) Expression efficiency of 3T3-L1 cells transfected with miR-328 mimics and inhibitor after 48 h (*n* = 4). (**C**–**H**) Simultaneous confirmation using qRT-PCR of CDK, CDK4 CCNB1, CCND1, CCNE1, and PCNA expression (*n* = 4). Note: QS represents Queshan Black pig; LW represents Large White pig. The GAPDH (for mRNA) and U6 (for miRNA) were used as normalization controls, * indicates *p* < 0.05, ** indicates *p* < 0.01, *** indicates *p* < 0.001.

The sequencing results showed a difference in the expression of miR-328 between Queshan Black and Large White pig. The qRT-PCR results showed that the expression of miR-328 in Queshan Black pigs was higher than that in Large White pigs, which is consistent with the sequencing results (Figure 7A). This study manipulated the expression of miR-328 in 3T3-L1 cells to study the effect of miR-328 on adipocytes. In the miR-328 mimics group, the expression of miR-328 increased (Figure 7B), whereas the expression of proliferation-related genes, such as CDK2, CDK4, CCNB1, CCND1, CCNE1, and PCNA, decreased. In the miR-328 inhibitor group, miR-328 was significantly inhibited, and contrasting results were obtained for CDK2, CDK4, CCNB1, CCND1, CCNE1, and PCNA (Figure 7C–H).

## 4. Discussion

As an essential dietary source for humans, meat is an important source of animal protein, and its quality is primarily reflected in its eating quality. Many factors can affect meat quality, such as genetic, muscle type, fat, and ultimate pH, as well as environmental influences [3].

Previous studies have shown that miRNAs play important roles in adipocyte proliferation and differentiation, thereby influencing meat quality traits. Although miRNAs consist of only 22 nucleotides, their mechanisms of action are diverse, which can affect phenotypic changes in a variety of ways. MiR-27a can inhibit adipocyte differentiation by targeting PPARγ [17]. MiR-27b was identified as a miRNA that regulates human adipogenesis and inhibits human adipocyte differentiation, and it regulates adipocyte differentiation by targeting PPARγ [18]. MiR-21 and miR-27b negatively regulate the expression of PPARα in the liver and other downstream genes; therefore, miR-21 and miR-27b could be used in managing lipid metabolism [19]. MiR-24-3p reverses the effects of ANXA6 on preadipocyte proliferation and differentiation by binding to the 3′UTR of ANXA6, which in turn affects the accumulation of intramuscular adipocytes [20]. MiR-208b regulates the transformation of different muscle fiber types by inhibiting Mettl8 expression [21]. MiR-100 reduces fetal bovine muscle satellite cell myogenesis and enhances intramuscular lipid deposition through the regulation of IGF1R, indicating its role in marbling characteristics and fat oxidation in the muscle of carnivores [22]. An analysis of miRNA expression profiles in duck adipocytes at 4 days of differentiation revealed that miR-214 could regulate duck fat deposition, targeting the 3′UTR of CPT2, decreasing the protein abundance of CPT2, and promoting lipid droplet formation [23]. MiR-152 affects pork quality by targeting and regulating the PKM gene, which encodes a key rate-limiting enzyme for glycolysis in pigs, thereby regulating lactate production [24]. MiR-195 reduces THRSP expression by targeting its 3′UTR, suggesting that miR-195 may inhibit lipid accumulation in adipocytes by regulating THRSP [25]. In this study, we used high-throughput sequencing with HiSeq technology to compare the differentially expressed miRNAs in two different breeds of pigs. This study will contribute to the understanding of the role of miRNAs in the regulation of pork quality.

MiRNAs regulate target gene functions by targeting the 3′UTR sequence of mRNAs; therefore, identifying the target mRNAs is important. In this study, miRNA target genes were predicted as the intersection of miRanda, PITA, and RNAhybrid. GO and KEGG enrichment analyses were performed on the set of miRNA target mRNAs based on the relationship between differentially expressed miRNAs and their target mRNAs. Thus, the present study enriched the differentially expressed miRNA target mRNAs to investigate miRNA functions, and the results showed that target mRNAs were mainly enriched in the AMPK signaling pathway [26], MAPK signaling pathway, PI3K-Akt signaling pathway, Jak-STAT signaling pathway, insulin signaling pathway, and sphingolipid signaling pathway. The enrichment analysis of the differentially expressed target mRNAs of differentially expressed miRNAs revealed that target mRNAs were enriched in sphingolipid metabolism, apoptosis, PI3K-Akt signaling pathway, and differentiation. The enrichment analysis of the negatively associated differentially expressed target mRNAs of differentially expressed miRNAs revealed that target mRNAs were enriched during focal adhesion, and basal transcription factors. Members of these signaling pathways could regulate adipocyte differentiation by affecting the expression of adipogenic transcription factors such as PIP2, CEBPA, PPARc, FABP4, and FASN [27,28,29], and cell proliferation and differentiation transcription factors such as LMNA and ACTB [30,31]. These potential target genes will contribute to the study of miRNA regulatory functions in adipogenesis, as well as cell proliferation and differentiation. In brief, this study identified 39 differentially expressed miRNAs in the longissimus dorsi of Queshan Black and Large White pigs, and miR-149, miR-204, and miR-328 have been investigated in adipogenesis and muscle development, which may play an important regulatory role in the formation of pork quality traits.

Based on functional enrichment analysis and miRNA–mRNA regulatory network construction, miRNAs may affect lipid deposition and skeletal muscle development, which has an impact on meat quality, and numerous studies have revealed that miRNAs play important biological roles in cancer, adipose, and myocytes. Zhao found that circRNA4557-miR149-5p might be involved in IMF deposition by constructing a network diagram of circRNA–miRNA interactions [32], and bta-miR-149-5p could significantly downregulate the expression level of lipogenic marker genes in bovine adipocytes, indicating that bta-miR-149-5p regulates lipid metabolism in bovine adipocytes [33]. In addition, bta-miR-149-5p inhibits bovine adipocyte proliferation and differentiation by targeting CRTC at the transcriptional and post-transcriptional levels [34]. The NEAT1-hsa-miR-204-5p-IGF1 axis may serve as a target for estradiol-leptin synergy in the uterine tissue of patients with obesity and as a biomarker to predict disease [35]. MiR-204 also affects obesity [36]. In particular, numerous studies have shown that miR-328 plays an important role in a variety of biological processes, such as cancer-related pathways and fat deposition processes, by regulating the expression of target genes. In hypoxia-induced pulmonary artery smooth muscle cells, miR-328-3p can regulate the expression of PFN1 and affect the viability and migration of pulmonary artery smooth muscle cells [37]. Moreover, miR-328 inhibits the proliferation and migration of pulmonary artery smooth muscle cells in the PDGFBB signaling pathway by targeting PIM-1 [38]. A comprehensive miRNA profile of seven stages in goats revealed that chi-miR-328-3p may be a key miRNA during muscle development in goats [39]. MiR-328 was found to be a regulator of brown adipose tissue differentiation, which modulates the commitment of preadipocytes [40]. In this study, miR-328 could affect the expression level of marker genes such as CDK2, CDK4, CCNB1, CCND1, CCNE1, and PCNA. The results showed that miR-328 inhibits 3T3-L1 cell proliferation, indicating that miR-328 may play an important role in lipid metabolism and fat deposition.

## 5. Conclusions

The present study identified 39 differentially expressed miRNAs in the longissimus dorsi of Queshan Black and Large White pigs. In addition, miR-149-PIK3CD, miR-204-STAT1, and miR-328-CSF1R/SMPD4 may be important to miRNA–mRNA interactions that affect cell proliferation and differentiation, as well as fat deposition and skeletal muscle development. Notably, miR-328 can affect the proliferation of 3T3-L1 cells, suggesting that miR-328 may have an important effect on meat quality traits.

## Figures and Tables

**Table 1:** Results of raw reads of Queshan Black (QS1, QS2, and QS3) and Large White pigs (LW1, LW2, and LW3) after quality control.

Sample	Raw Reads	*n*% > 10%	Low Quality/%	Q30/%	GC Content/%	Clean Reads
QS1	30787856	0.00	0.07	99.00	44.99	30128527 (97.86%)
QS2	26744869	0.00	0.06	99.20	45.50	26165135 (97.83%)
QS3	33952308	0.00	0.07	98.97	45.44	33181446 (97.73%)
LW1	27521932	0.00	0.11	99.01	45.42	27207230 (98.86%)
LW2	25071908	0.00	0.12	98.79	45.20	24612756 (98.17%)
LW3	24938800	0.00	0.11	99.09	45.41	24540296 (98.40%)

**Table 2:** Alignment results of Queshan Black (QS1, QS2, and QS3) and Large White pigs (LW1, LW2, and LW3) using the specified Sscrofa11.1/susScr11 as the reference genome.

Sample	Total sRNA	Mapped sRNA	Mapped sRNA (+)	Mapped sRNA (−)
QS1	29506123	28479022 (96.52%)	21790911 (73.85%)	6688111 (22.67%)
QS2	25548730	24589772 (96.25%)	19112359 (74.81%)	5477413 (21.44%)
QS3	32472797	31216845 (96.13%)	23834912 (73.40%)	7381933 (22.73%)
LW1	26511199	25669736 (96.83%)	20633956 (77.83%)	5035780 (18.99%)
LW2	23789195	22683871 (95.35%)	16728401 (70.32%)	5955470 (25.03%)
LW3	23893307	22661469 (94.84%)	16207603 (67.83%)	6453866 (27.01%)

**Table 3:** Statistics of miRNA target gene prediction results.

Type	Number of miRNA	Target mRNA	Target Gene
All miRNA	418	20,375	10,428
Differentially expressed miRNA	39	5234	3064

## Data Availability

All raw data of high-throughput sequencing were deposited to the National Genomics Data Center (NGDC, https://bigd.big.ac.cn, accessed on 19 September 2021) with the dataset accession number CRA005025.

## Supplementary Materials

The following supporting information can be downloaded at: https://www.mdpi.com/article/10.3390/life12111814/s1, Figure S1: GO of DEmiRNA target mRNA; Figure S2: KEGG of DEmiRNA target mRNA; Figure S3: GO of DEmiRNA target DEmRNA; Figure S4: KEGG of DEmiRNA target DEmRNA; Figure S5: GO of DEmiRNA target negative mRNA; Figure S6: KEGG of DEmiRNA target negative mRNA; Table S1: GO of DEmiRNA target mRNA; Table S2: GO enrichment of DEmiRNA target mRNA; Table S3: KEGG of DEmiRNA target mRNA; Table S4: Pathway Enrichment of DEmiRNA target mRNA; Table S5: GO of DEmiRNA target DEmRNA; Table S6: GO enrichment of DEmiRNA target DEmRNA; Table S7: KEGG of DEmiRNA target DEmRNA; Table S8: Pathway Enrichment of DEmiRNA target DEmRNA; Table S9: GO of DEmiRNA target negative mRNA; Table S10: GO enrichment of DEmiRNA target negative mRNA; Table S11: KEGG of DEmiRNA target negative mRNA; Table S12: Pathway Enrichment of DEmiRNA target negative mRNA.
